# In vitro toxicological evaluation of surgical smoke from human tissue

**DOI:** 10.1186/s12995-018-0193-x

**Published:** 2018-04-02

**Authors:** Jennifer D. Sisler, Justine Shaffer, Jhy-Charm Soo, Ryan F. LeBouf, Martin Harper, Yong Qian, Taekhee Lee

**Affiliations:** 1 Pathology and Physiology Research Branch, Health Effects Laboratory Division, National Institute for Occupational Safety and Health, Centers for Disease Control and Prevention, 1095 Willowdale Road, Morgantown, West Virginia 26505 USA; 2 Exposure Assessment Branch, Health Effects Laboratory Division, National Institute for Occupational Safety and Health, Centers for Disease Control and Prevention, 1095 Willowdale Road, Morgantown, West Virginia 26505 USA; 3 Field Study Branch, Respiratory Health Division, National Institute for Occupational Safety and Health, Centers for Disease Control and Prevention, 1095 Willowdale Road, Morgantown, West Virginia 26505 USA; 4 Zefon International, Inc., 5350 SW 1st Lane, Ocala, FL USA; 5 Department of Environmental Engineering Sciences, University of Florida, Gainesville, FL USA

**Keywords:** Surgical smoke, Toxicology, Healthcare workers

## Abstract

**Background:**

Operating room personnel have the potential to be exposed to surgical smoke, the by-product of using electrocautery or laser surgical device, on a daily basis. Surgical smoke is made up of both biological by-products and chemical pollutants that have been shown to cause eye, skin and pulmonary irritation.

**Methods:**

In this study, surgical smoke was collected in real time in cell culture media by using an electrocautery surgical device to cut and coagulate human breast tissues. Airborne particle number concentration and particle distribution were determined by direct reading instruments. Airborne concentration of selected volatile organic compounds (VOCs) were determined by evacuated canisters. Head space analysis was conducted to quantify dissolved VOCs in cell culture medium. Human small airway epithelial cells (SAEC) and RAW 264.7 mouse macrophages (RAW) were exposed to surgical smoke in culture media for 24 h and then assayed for cell viability, lactate dehydrogenase (LDH) and superoxide production.

**Results:**

Our results demonstrated that surgical smoke-generated from human breast tissues induced cytotoxicity and LDH increases in both the SAEC and RAW. However, surgical smoke did not induce superoxide production in the SAEC or RAW.

**Conclusion:**

These data suggest that the surgical smoke is cytotoxic in vitro and support the previously published data that the surgical smoke may be an occupational hazard to healthcare workers.

## Background

Approximately 20 million Americans undergo surgery with general anesthesia each year [1]. Nowadays, electrocautery, laser ablation, and ultrasonic scalpel dissection are widely recognized as major advances in surgical technique and are increasingly being used for tissue cutting and hemostasis [2]. Surgical incision and dissection with electrocautery, laser and ultrasonic scalpel are used to cut tissue and decrease bleeding through coagulating small blood vessels. The key feature of these techniques is to heat tissue to high temperatures that burn and rupture cellular membranes and other structures. However, the breakdown of cellular membranes and other tissue structures generates many biological by-products that mix with chemical compounds used during surgery, which form smoke due to the high temperatures during the surgical procedures. The released surgical smoke contaminates the air with many chemical compounds as by-products of tissue damage, as well as biological materials, including potentially infectious agents [3]. Several studies have found that the complex mixture of surgical smoke contains both chemical pollutants and biological hazards [2, 4–7]. The composition of the surgical smoke varies based upon the type of surgery; however, the following chemical components have been found to be common to most surgeries: acetaldehyde, acrolein, acetonitrile, benzene, hydrogen cyanide (HCN), polyaromatic hydrocarbons (PAHs), styrene, toluene, and xylene [2, 4, 8–11]. Animal studies have shown that rats exposed to smoke from pigskin showed congestive pneumonia, bronchiolitis and emphysema [12] and sheep exposed to smoke from sheep bronchial tissue showed a decrease of arterial PO_2_ (hypoxia), depressed tracheal mucus velocity and severe inflammation with dramatic increases of inflammatory cells [13]. A survey showed that operating room nurses reported respiratory problems including nasal congestion, increased coughing, allergies and sinus infections or problems and the prevalence for the nurses was greater than the prevalence in the US [14]. In addition, Health Hazard Evaluations (HHEs) by the National Institute for Occupational Safety and Health (NIOSH) on surgical smoke exposure have been requested repeatedly over the past decades suggesting that OR personnel are experiencing adverse reactions to exposure to surgical smoke [15–19]. Surgical smoke has also been shown to contain several known carcinogenic compounds. NIOSH reported on the mutagenicity of surgical smoke generated in reduction mammoplasty procedures [20]. Recently, it was found that surgical smoke has ultrafine particles that are in the range of 9–81 nm depending on the type and the duration surgery [3, 21]. Ultrafine particles in the surgical smoke have the ability to reach the alveolar region of the lung and cause pulmonary inflammation or disease [22, 23].

Human toxicological response to surgical smoke has not been studied in detail. An in vitro study has shown that surgical smoke collected into cell culture media from cutting porcine liver using an electro-surgical hook knife caused a toxic effect on human breast cancer cells (MCF-7) using the clonogenic assays [24]. Other in vitro studies have shown that electrocautery of cultured retrovirus infected melanoma cells produced airborne viable retrovirus particles and electrocautery of a pellet of melanoma cells released viable melanoma cells [25, 26]. However, another group has shown that tumor ablation with ultrasonically activated scalpel or electrocautery does not release viable airborne cancer cells [27]. Although transmission risk of human virus or cancer cells by inhalation of surgical smoke is not clear, the concerns have been raised that human cancer cells, viruses including human immunodeficiency virus and other pathogens could become airborne through the use of surgical devices [12]. Moreover, the toxicological effects of other biological products and chemical pollutants in surgical smoke have not been fully determined. It is important to better understand the toxicological effects of surgical smoke because surgical smoke could create an occupational hazard to operating room staff. It is important to identify the risk of surgical smoke to guide the installation of the proper protection procedures and devices in surgical rooms. Recently, a survey was performed to determine if the correct engineering controls were being used to protect healthcare workers from exposure to surgical smoke [28]. Their survey concluded that a majority of surgical rooms did not have proper local exhaust ventilation (LEV) because the installation of LEV in these surgical rooms was not considered in their design. Moreover, those surgical rooms without LEV also did not have respirators and the healthcare workers only used surgical masks [28]. Therefore, the healthcare workers who work in these surgical rooms have no proper protection procedures and devices to prevent the potential exposure to surgical smoke.

While several in vivo and in vitro studies suggest the toxicity of surgical smoke using cultured cells, virus or pig skin, information concerning the toxicological effects of surgical smoke generated from human tissue has been lacking. In this study, the cellular toxicity of surgical smoke of human tissue was assessed. Human breast tissues were cut using an electrocautery surgical device and the surgical smoke was collected in real time into cell culture media, followed by exposure to human small airway epithelial cells (SAEC) and mouse macrophages (RAW). The chemical properties and the in vitro toxicity of surgical smoke generated with real human tissues were analyzed.

## Methods

### Surgical smoke generation and collection

Fresh human breast tissues obtained from the West Virginia University (WVU) tissue bank were used to generate surgical smoke in unoccupied operating rooms at WVU Ruby Memorial hospitals. The tissues were obtained within 3 h after breast reduction surgeries. Surgical smoke was generated with an electrocautery surgical device (model: ForceFX, Valleylab, Boulder, CO, USA; output power of cut and coagulate was set at 35 watts; cut and coagulation modes mixed use) for 15 min. The smoke was collected with three autoclaved BioSamplers® (SKC Inc., Eighty Four, PA, USA) for each generation and each sampler was loaded with 2 mL of cell medium, either Dulbecco’s Modified Eagle Medium (DMEM) or Small Airway Epithelial Cell growth medium (SABM). Inlets of the BioSamplers® were maintained within 5 cm of electrocautery interaction site, as this represents the worst-case situation when OR personnel lean in over the patient during surgery. Samples were collected at 12.5 l/min over 15 mins to collect the smoke visually observed from the interaction site. A total of 24 surgical smoke generations (each 15 mins generation) were conducted in 6 different sampling sessions. A total of 33 samples, and 39 samples, were collected using BioSamplers® loaded with DMEM and SABM, respectively, along with 33 background samples (air sampling with the BioSamplers® was conducted without generation of surgical smoke for each cell medium) and 11 field blank samples (no sampling).

### Air sampling and sample analysis

#### Direct reading instrument measurement

Particle number concentration and particle distribution were measured in real time with direct reading instruments. The number concentration of particles in the size range of 0.01–1.0 μm were measured using a condensation particle counter (CPC, model 3007, TSI Inc., Shoreview, MN, USA) every second. The particle size distribution of ultrafine particles was measured by a Scanning Mobility Particle Sizer (SMPS, model 3034, TSI Inc., size range from 10 to 414 nm) or Nanoscan SMPS nanoparticle sizer (Model 3910, TSI Inc., size range from 11.5 to 365.2 nm), but not for all of the generations due to instruments availability.

#### Scanning electron microscope analysis

A field emission scanning electron microscope (SEM; model S-4800-2, Hitachi High Technologies America Inc.) was utilized to characterize airborne particles collected in cell medium. Surgical smoke sample collected in the DMEM media sample was filtered onto a polycarbonate filter (pore size 0.45 μm, 25-mm diameter) and desiccated and sputter-coated with gold and platinum. SEM analysis for the SABM media could not be conducted because the media was too dense to be filtered.

#### VOCs sampling and analysis

Volatile organic compounds were collected using evacuated canisters following NIOSH draft canister method for VOCs in air [29]. Area sampling was conducted using 6 L (SilicoCan, Restek Corporation, Bellefonte, PA, USA) or 600 mL (Silonite® miniCans with Micro-QT Valves, Entech Instrument Inc., Simi Valley, CA, USA) canisters. Critical orifice (Restek Corporation) or sapphire restrictors (Restek Corporation) were utilized to maintain flow rates of the passively sampling evacuated canisters. The flow rates of 6 L and 600 mL of the canisters were 100 or 35 cm^3^/min, respectively. A grab sampling technique of filling canisters within 1 min was utilized to collect VOCs within 5 cm from electrocautery interaction site. Area sampling technique was used in sampling #1, #2 and #3 and grab sampling technique was in sampling #4, #5 and #6. The air samples were analyzed using a pre-concentrator (7200, Entech Instrument Inc.) and gas chromatography-mass spectrometry (Agilent Technologies, Inc., Santa Clara, CA, USA) system in accordance with the methodology presented in a recently published method validation study [29, 30]. The study quantified VOCs associated with healthcare settings including: α-pinene, acetone, benzene, chloroform, ethanol, ethyl benzene, hexane, isopropyl alcohol, *d*-limonene, m, p-xylene, methyl methacrylate, methylene chloride, o-xylene, and toluene. Three additional VOCs were added to the target list for this study: acetaldehyde, acetonitrile, and styrene. Other VOCs were qualitatively identified by comparing their mass spectra to the NIST 2008 Mass Spectral Library. They were included if the quality factor of comparison was greater than 90%.

#### Head space analysis

In order to quantify dissolved VOCs in the cell media, head space analysis was conducted although it is not a direct measurement of the cell medium. One mL sample from each blank and the DMEM and SABM containing surgical smoke was transferred into a sealed 40 mL amber volatile organic analysis vial and allowed to rest for 24 h at room temperature (21 °C) in the laboratory. Then 2 mL of headspace air was transferred to a 450 mL canister and pressurized to approximately 1.5 times of atmospheric pressure. Using the canister analysis system, the concentrations were calculated in parts per billion (ppb) of analytes in the headspace. VOCs concentration from the blank of each medium was subtracted from the surgical smoke samples. Two samples were collected for each cell medium in sampling #4 and #6.

### Cell culture--human small airway epithelial cells

Dr. Tom K. Hei at Columbia University (New York, NY, USA) provided the human small airway epithelial cell lines (SAEC) and these were cultured as previously described [31]. Briefly, the SAEC were maintained in serum free SABM with the following supplements: bovine pituitary extract, hydrocortisone, human epidermal growth factor, epinephrine, transferrin, insulin, retinoic, triiodothyronine, gentimicin amphotericin-B, and bovine serum free albumin-fatty acid free which are all provided as a bullet kit from the manufacturer (Lonza Inc., Allendale, NJ, USA). The SAEC were plated at the appropriate density and allowed to fully attach for 24 h at which point the media was changed. At 48 h after seating the cells, the media was changed to SABM free of supplements. For the last 24 h of the assay, the surgical smoke SABM was added to the SAEC before being assayed.

### Cell culture--RAW 264.7 mouse macrophage

Mouse macrophage cells RAW 264.7 (RAW) were purchased from American Type Culture Collection (ATCC) (Manassas, VA, USA) and were maintained following source company guidelines. The RAW cells are cultured in DMEM with L-glutamine (Lonza, Allendale, NJ, USA) with the following supplements: 10% feta bovine serum (FBS) (Flowery Branch, GA, USA) and 5% penicillin/streptomycin (Lonza, Allendale, NJ, USA). RAW cells were plated at the correct density for each experiment and after 24 h were treated with the surgical smoke-DMEM for an additional 24 h before assayed.

### Cytotoxicity of surgical smoke in vitro

SAEC were plated at 1.5 × 10^4^ cells per well and the RAW cells were plated at 1.0 × 10^4^ cells per well (BD Biosciences, San Jose, CA, USA). Changes in cellular proliferation after a 24 h treatment with surgical smoke were assayed using the 3-(4,5-dimethylthiazol-2-yl)-5-(3-carboxymethoxyphenyl)-2-(4-sulfophenyl)-2H-tetrazolium (MTS) assay (Cell Titer 96 ® Aqueous One Solution Cell Proliferation Assay kit (Promega, Madison, WI, USA) following the manufacturer’s guidelines.

### Lactate dehydrogenase production in vitro after treatment with surgical smoke

To measure the membrane integrity of the SAEC or RAW cells, 1.5 × 10^4^ SAEC and 1.0 × 10^4^ RAW per well were plated in 96 well plates and assayed after a 24 h treatment with surgical smoke using the CytoTox-ONE™ Homogeneous Membrane Integrity Assay (Promega, Madison, WI, USA) following the manufacturer’s protocol.

### Reactive oxygen species (ROS) production after treatment with surgical smoke

SAEC and RAW were plated at 1.5 × 10^4^ and 1.0 × 10^4^ cells per well in a 96 well plate, respectively. For the last thirty minutes of a 24 h treatment with surgical smoke, the cells were dosed with 5 μM 2′,7′-dichlorofluorescin diacetate (DCFDA) (Life Technologies, Carlsbad, CA) in dimethyl sulfoxide (DMSO). The plate was then read at 492 nm and 517 nm wavelengths in a microplate reader to analyze the ROS production.

## Results

### Airborne particles and VOCs concentrations of surgical smoke

The generation rate of the smoke may be different not only from real surgeries but also between each 15 min generation. It was technically difficult to quantify collected particles and dissolved VOCs in cell medium; therefore, airborne particles and VOCs concentrations during generations were reported together with head space analysis of VOC samples dissolved in cell culture media, which approximates real human exposure in healthcare environment.

**Fig. 1:**
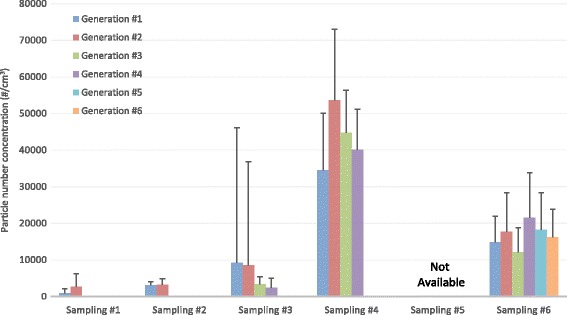
Average and standard deviation of particle number concentration in each surgical smoke generation (15 min) measured by a condensation particle counter (particle size range 0.01–1.0 μm)

**Fig. 2:**
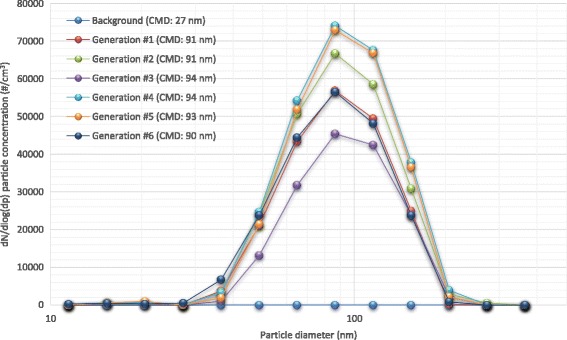
Particle size distribution of surgical smoke measured with a Nanoscan Scanning Mobility Particle Sizer nanoparticle sizer (Sampling #6). CMD is counter median diameter

**Fig. 3:**
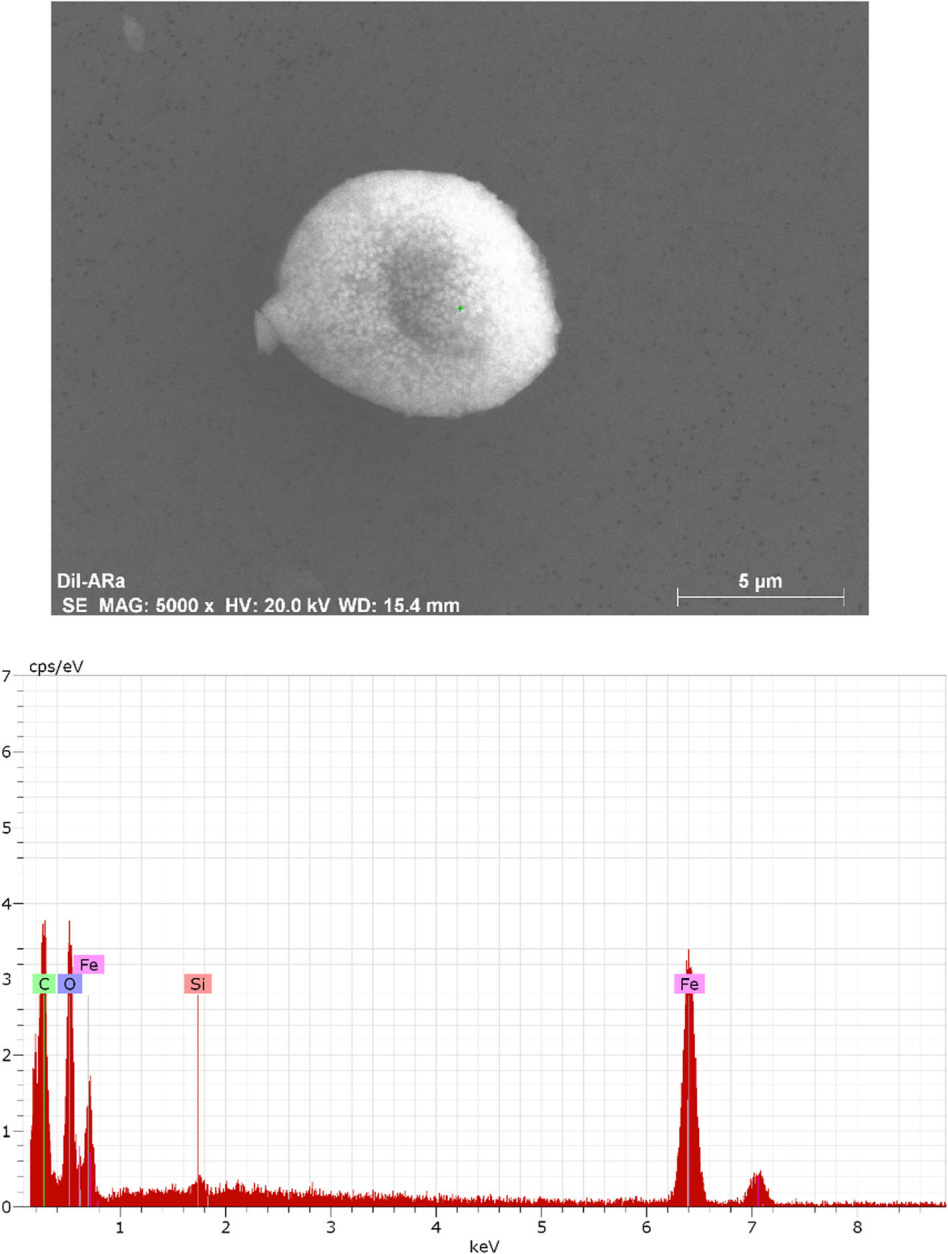
Particles collected onto cell medium and filtered onto a polycarbonate filter with scanning electron microscope image along with X-ray counts

Average particle number concentrations in operating room background (30 min) and each surgical smoke generation (15 min) measured by a condensation particle counter (particle size range 0.01–1.0 μm) for each sampling are shown in Fig. 1. The average background particle concentration ranged from 1 to 1600 particles/cm^3^. Due to a malfunction of the CPC, particle concentrations in sampling #5 were not obtained. The average particle number concentrations ranged from 900 (Generation #1 in Sampling #1) to 54,000 (Generation #2 in Sampling #4) particles/cm^3^. Ratios of average of 15 min surgical smoke generation to average of background particle number concentration ranged from 2 (sampling #2 and #5) to 5200 (sampling #1; high ratio due to low background concentration ≈ 1 particle/cm^3^). An example of the particle size distributions in Sampling #6 is shown in Fig. 2. Average and standard deviation of the count median diameters was 92 ± 1.7 nm. Background particle distribution was significantly different from particle distributions of all generations (*p* < 0.05) and generation #3 and #4 showed significant difference in accordance with a two-way ANOVA using Tukey’s Studentized Range test (SAS Ver. 9.4, SAS Institute Inc., Cary NC).Qualitative SEM analysis was conducted with samples from filtered DMEM media sample and an example of a particle is shown in Fig. 3 along with un-calibrated elemental counts by energy-dispersive x-ray spectrometer. The particles were amorphous shape and had similar X-ray element distributions. Airborne particles in the micrometer size-range were observed but particles in nanometer sizes that were detected with direct reading instruments were not identified.

Average concentrations (μg/m^3^; average of all canister samples (*n* = 36), area and grab sampling, from each sampling session) of VOCs sampled with evacuated canisters for each sampling are shown in Table 1 along with average background concentrations. Higher concentrations of VOCs were found with grab sampling than area sampling. All targeted 17 different VOCs were detected in most of sampling sessions. Higher concentration of the VOCs were found in sampling with surgical smoke compared to background concentration. Acetaldehyde, ethanol and isopropyl alcohol were predominantly detected in every sample with high concentrations (up to 14,000 μg/m^3^ of isopropyl alcohol) compared to other VOCs. Tentatively identified VOCs found in background air sampling with the NIST 2008 Mass Spectral Library(quality factor > 90%) were propene, 2-propanol, 2-propanone, 3-buten-2-one, acetone, acetonitrile, butane 2,2-dimethyl-, ethanol, isoflurane, pentane 2-methyl-, phenol, sevoflurane. Tentatively identified VOCs found in air sampling with surgical smoke generation were 1-propene, 2-methyl-; 1 butanal, 3-methyl-; 1 butanal, 2-methyl-; propene; propyne; 1,4-pentadiene; 1,3-butadiene, 2-methyl-; 2-propenenitrile; 1,3-butadiene; 1-buten-3-yne, 2-methyl-; 1-hexene; 1-heptene; *trans*-1-butyl-2-methylcyclopropane; 2-butenenitrile; 3-butenenitrile; pyridine; pyrrole; propanal, 2-methyl-; 1,3-pentadiene; 1,3-cyclopentadiene; cyclopentene; 2-propenal; *cis*-1-butyl-2-methylcyclopropane; cyclopropane, 1-ethyl-2-heptyl-; cyclopropane, ethylidene-; pentane; 2-methyl-1-butene; 1-decanol; 2,3-pentadiene; 4-methyl-1,3-pentadiene; 1-pentene, 2-methyl-; 1-methylcyclopropene; 1,3-pentadiene, (E)-; 1H-pyrrole.

**Table 1:** Average concentrations (μg/m^3^) of volatile organic compounds from surgical smoke generation

	REL (μg/m3)	Background	Sampling #1	Sampling #2	Sampling #3	Sampling #4	Sampling #5	Sampling #6
Acetaldehyde	lowest feasible	12	13	17	1200	940	630	2100
Acetone	590,000	21	30	41	38	150	81	170
acetonitrile	34,000	5	1	12	440	410	130	570
α-pinene	–	4	–	–	5	4	5	9
Benzene	3190	2	1	–	120	560	220	130
Chloroform	9780	–	–	–	–	–	10	–
d-Limonene	–	7	–	11	10	12	10	18
Ethanol	1,900,000	450	37	1200	290	810	230	1100
Ethylbenzene	435,000	2	–	5	51	39	15	23
Isopropyl Alcohol	980,000	870	110	1000	380	1900	400	14,000
m,p-Xylene	435,000	3	4	4	21	13	10	3
Methyl methacrylate	410,000	4	–	–	8	–	11	–
Methylene chloride	lowest feasible	–	–	–	–	–	7	–
n-Hexane	180,000	3	–	–	22	85	42	33
o-Xylene	435,000	0	6	–	4	6	6	2
Styrene	215,000	–	–	–	91	49	17	31
Toluene	375,000	4	4	6	90	190	72	99

-: below detection limit or not detected

REL: Recommended exposure limits from National Institute for Occupational Safety and Health

Head space analysis results are shown in Table 2. Less VOCs were detected by the head space analysis compared to canister sample analysis due to different solubility and volatility of the VOCs while some VOCs were higher concentrations with large variation.

**Table 2:** Volatile organic compounds concentrations from head space analysis

Volatile Organic Compounds	REL (μg/m^3^)	Dulbecco’s Modified Eagle Medium (μg/m^3^)		Small Airway Epithelial Cell growth medium (μg/m^3^)	
		Test 1	Test 2	Test 1	Test 2
Acetaldehyde	lowest feasible	1700	4000	1700	2900
Acetone	590,000	*	*	540	*
Acetonitrile	34,000	420	–	440	–
alpha-Pinene	–	–	–	–	–
Benzene	3190	–	160	31	260
Chloroform	9780	–	–	–	–
D-Limonene	–	–	–	–	–
Ethanol	1,900,000	*	*	490	5800
Ethylbenzene	435,000	*	–	700	98
Isopropyl Alcohol	980,000	25,000	47,000	4400	37,000
m,p-Xylene	435,000	*	–	550	140
Methyl Methacrylate	410,000	–	–	–	–
Methylene Chloride	lowest feasible	–	–	–	–
n-Hexane	180,000	–	–	–	–
o-Xylene	435,000	–	–	–	–
Styrene	215,000	–	–	–	–
Toluene	375,000	*	–	66	110

-: below detection limit or not detected

*: the concentration from surgical smoke is smaller than blank cell medium

REL: Recommended exposure limits from National Institute for Occupational Safety and Health

### Cytotoxicity of surgical smoke

**Fig. 4:**
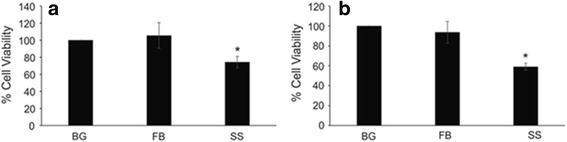
Surgical Smoke Induced Cytotoxicity. (**a**) SAEC and (**b**) RAW were dosed with surgical smoke for 24 h and then cytotoxicity was measured using an MTS assay. The t-test was applied. Values represent mean ± standard error. *n* = 4 independent biological replicates. * indicates *p* < 0.05 compared to field blank (FB)

It has been suggested that surgical smoke is toxic both in vitro and in vivo [12, 24]. Because the surgical smoke has ultrafine particles, the pulmonary alveolar region could potentially be affected; therefore, cytotoxicity was measured in human small airway epithelial cells (SAEC). Macrophage cells are the first line of defense against any foreign material that enters the body; therefore it is of importance to measure the cytotoxicity of RAW cells. SAEC and RAW cells were dosed with surgical smoke collected into the respective media or a background or field blank sample control using an MTS assay. Surgical smoke caused approximately 25% cell death in the SAEC and 40% in the RAW cells compared to background and field blank (Fig. 4). Both of these changes were statistically significant (*p* < 0.05) when compared to either the background or field blank samples. This would suggest that the cell death seen is due to the surgical smoke generated from the human breast tissue. Taken together this data would suggest that the surgical smoke is more cytotoxic to the RAW cells when compared to the SAEC cells.

### Production of lactate dehydrogenase (LDH) by surgical smoke

**Fig. 5:**
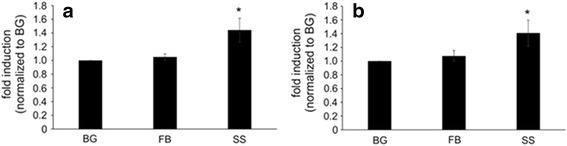
Surgical Smoke Induced Lactate Dehydrogenase. (**a**) SAEC and (**b**) RAW cells were dosed with surgical smoke for 24 h and analyzed for the production of LDH. The t-test was applied. Values represent mean ± standard error. n = 4 independent biological replicates. * indicates *p* < 0.05 compared to field blank (FB)

Ultrafine particles have been shown to induce the production of lactate dehydrogenase (LDH) which is a measurement of cellular membrane damage [32, 33]. To analyze the integrity of the cellular membrane, lactate dehydrogenase (LDH) levels released into the cell culture media after a 24 h treatment of surgical smoke were analyzed. Figure 5 shows that both SAEC and RAW cells produced significantly higher levels of LDH after a 24 h dose of surgical smoke compared to both the background and field blank samples. This would suggest that the surgical smoke caused membrane damage in both the SAEC and RAW cells.

### Production of ROS by surgical smoke

**Fig. 6:**
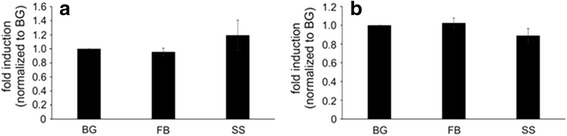
ROS Induced by Surgical Smoke. (**a**) SAEC and (**b**) RAW dosed cells were analyzed for DCFDA after a 24 h treatment with surgical smoke. The t-test was applied. Values represent mean ± standard error. n = 4 independent biological replicates

ROS production has been shown to be a key mechanism to lead to cytotoxicity both in vitro and in vivo [34, 35]. Therefore, it was of interest to determine if surgical smoke induced the production of ROS. To determine if ROS was produced, 5 μM DCFDA was added to the SAEC and RAW cells for the last 30 min of a 24 h exposure of surgical smoke. If free radicals are present, DCFDA is oxidized and cleaved into DCF which fluoresces. As shown in Fig. 6, neither SAEC nor RAW cells shown significant increases in ROS production compared to the background or field blanks. This would suggest that the molecular mechanism leading to the cytotoxicity seen by the MTS and LDH assay is independent to superoxide radicals. While there was a trend that SAEC cells did produce ROS compared to the controls, it was not significant and would need further analysis to determine if it was a true induction.

## Discussion

The levels of smoke generated in this study most likely represent the worst possible case of exposure, when OR personnel lean in over the patient during surgery, which is likely only for brief periods of time during the workday. Particle concentrations measured with direct reading instruments were comparable to previous studies. Average particle number concentrations measured with a CPC in six different surgeries without local exhaust ventilation (LEV) control ranged from 74 to 12,200 particles/cm^3^ with a range of 2–490,000 particles/cm^3^ [36]. Ragde et al. reported ultrafine particle exposure levels in five different surgeries when LEV was utilized and a maximum peak level was 272,000 particles/cm^3^ while average levels were between 300 and 3900 particles/cm^3^ [3]. The average particle number concentration from the present study ranged from 900 to 54,000 particles/cm^3^. Elmashae et al. (2018) simulated OR facility to assess surgical smoke (from lamb muscle tissue) exposure of unprotected OR workers and reported that peaks of the particle size distribution were between 60 and 150 nm, which is consistent with the present study (count median diameters 92 ± 1.7 nm; Fig. 2) [37]. The differences in particle concentrations between the studies might be attributable to surgery type, tissue types, power level of electrocautery, ventilation rate of the operation room, surgeon’s technique, utilizing LEV system, etc. Personal VOC exposure levels from full-shift sampling were lower than the concentrations found during the 15 min or 1 mins sampling of the present study because the majority of a workers’ shift would not involve electrocauterization. For instance, LeBouf et al. [29] reported VOCs exposure levels in 14 occupations of healthcare workers and personal exposure levels (μg/m^3^) of surgical technologists (geometric mean) were 1031 (ethanol), 1077 (isopropyl alcohol), 0.98 (benzene), 112 (toluene), < 0.16 (ethylbenzene), 1.8 (m,p-xylene), < 0.19 (o-xylene), 72 (acetone), 0.14 (hexane), < 0.17 (methyl methacrylate), 3.1 (methylene chloride), 0.18 (chloroform). Relatively high levels of ethanol and isopropanol are to be expected in hospital settings and were also noted in our background results.

Large number concentration of ultrafine particles measured by direct reading instrument were not identified by SEM analysis in this study. A companion study compared airborne particle and VOCs levels with and without LEV controls and utilized the same procedure and human tissues as the present study to generate the surgical smoke [38]. The study conducted qualitative scanning electron microscope (SEM) analysis using an inhalable sampler (IOM sampler) to detect airborne surgical smoke particles along with elemental counts by energy-dispersive x-ray. The finding from that study was comparable to the present study. The particles were amorphous in shape and had similar elemental distribution. Airborne particles in micrometer sizes were observed in 45 samples but ultrafine particles were not identified. Most of the airborne particles appeared to be water or steam from cellular fluid from adipose tissues as previously noted [5, 39]. Kunachak and Sobhon reported SEM images of smoke particles generated with a carbon dioxide laser from papillomatous tissue and particles sizes ranged from 0.5 to 27 μm [39]. Particles smaller than 500 nm were not found.

The results of the MTS assays in this study suggest that the surgical smoke is cytotoxic to both the SAEC and RAW cell lines, but to varying degrees. One possible reason for the difference seen in the levels of cytotoxic effect on the cell lines is because the molecular mechanisms related to cell death in each cell line are different. To explain the cytotoxic effects of the surgical smoke in SAEC and RAW, LDH was measured and shown to be elevated in both the SAEC and RAW which correlates with the MTS assay. This data would suggest that the surgical smoke is a potential health hazard to individuals during surgery, which correlates with previously published data [24]. The cytotoxicity could be related to the exposure to the VOCs dissolved in the culture media listed in Tables 1 and 2 or it could possibly be due to the surgical smoke collected in the culture media. The results of Tables 1 and 2 demonstrated that all the concentrations of the VOCs are below the NIOSH RELs. Thus, it is possible that the VOCs may not play a major role in the surgical smoke-induced cellular toxicity. To clarify this point, additional studies will need to be performed to separate the particles and VOCs. In the present study, concentrations of airborne VOCs have been determined (Table 1) whereas the exact concentrations of the VOCs in cell culture medium have not determined. The head space analysis was applied to indirectly measure the concentrations of the dissolved VOCs in the cell culture medium. Therefore, it is possible that some disparities might exist between our measured concentrations of VOCs and the real concentrations in the cell culture medium due to the different solubility and the rate of evaporation of each VOC. In the future, the direct measurement of the VOC concentration in the cell culture medium needs to be developed to identify the contribution of the VOCs in surgical smoke-induced cellular toxicity.

Oxidative stress can mediate molecular mechanisms of cytotoxicity in particulate exposure [35]. Oxidative stress is a term that encompasses superoxide radicals (O_2_^−^), hydrogen peroxide (H_2_O_2_), hydroxyl radical (^−^OH) and peroxynitrite (ONOO^−^) [34]. To determine if oxidative stress played a role in the cytotoxicity that is seen in the SAEC and RAW after treatment with surgical smoke, DCFDA was used to measure ROS production. Based upon the results, ROS production was not elevated in either the SAEC or RAW suggesting the cytotoxicity seen from surgical smoke is independent of ROS production. To investigate other possible mechanisms, further experiments would be needed. Ultrafine particles are also deposited in the nasal airways and this may be worth further investigation given the reporting of sino-nasal symptoms [14].

## Conclusions

This study collected surgical smoke (particulate and VOCs) into cell culture media in a real-time exposure setting that allowed for characterization of the particles and analysis of the VOCs released into the air, and the analysis of the toxic effects of the smoke in an in vitro model. The results indicate that the surgical smoke is toxic in both the SAEC and RAW although to varying degrees. This data again is consistent with previously published data. To fully understand the toxic effect of the surgical smoke, further experiments would need to be performed in vitro to determine if the particles or the VOCs (or the combination) are the cause of the identified cytotoxicity and also to perform in vivo testing.

## Abbreviations

CPC: condensation particle counter
DCFDA: 2′,7′-dichlorofluorescin diacetate
DMEM: Dulbecco’s Modified Eagle Medium
DMSO: Dimethyl sulfoxide
FBS: fetal bovine serum
HCN: hydrogen cyanide
HHEs: Health Hazard Evaluations
IOM: Institute of Occupational Medicine
LDH: Lactate dehydrogenase
LEV: Local exhaust ventilation
MTS: 3-(4,5-dimethylthiazol-2-yl)-5-(3-carboxymethoxyphenyl)-2-(4-sulfophenyl)-2H-tetrazolium
NIOSH: National Institute for Occupational Safety and Health
PAHs: Polyaromatic hydrocarbons
ppb: Parts per billion
RAW: RAW 264.7 mouse macrophages
ROS: Reactive oxygen species
SABM: Small Airway Epithelial Cell growth medium
SAEC: Small airway epithelial cells
SEM: Scanning electron microscope
SMPS: Scanning mobility particle sizer
VOCs: Volatile organic compounds
WVU: West Virginia University
